# Meaning and Significance of “Alkalization Therapy for Cancer”

**DOI:** 10.3389/fonc.2022.920843

**Published:** 2022-07-14

**Authors:** Hiromi Wada, Reo Hamaguchi, Ryoko Narui, Hiromasa Morikawa

**Affiliations:** Japanese Society on Inflammation and Metabolism in Cancer, Kyoto, Japan

**Keywords:** cancer, metabolism, tumor microenvironment, alkalization therapy, urine pH, bicarbonate

## Abstract

**Objectives of the Study:**

Our research aims to answer the following questions. Can cancer progression be stopped by changing the body condition of person with cancer? Can cancer be cured?If cancer progression can be stopped, what is the underlying mechanism?

**Theoretical Rationale for Alkalization Therapy:**

Almost 70 years ago, Goldblatt H. & Cameron G. reported on the idea of alkalization therapy. Before that, Otto Warburg had been studying the metabolism of cancer and had discovered the essential nature of cancer. He published a review in Science in 1956 under the title “On the origin of cancer cells”. From his phenomena described above, we established the theoretical rationale for alkalization therapy, based on the question of “How does cancer form and what is its nature”?

**Limitations of Deductive Methods and Inductive Approaches:**

In this paper, we describe a method to reconstruct the limitations and weaknesses of modern cancer medicine as Science-based Medicine using an inductive method, and to present a new vision of cancer therapy. How should we treat cancer? (Case presentation): Using a specific clinical case, we present patients in whom were successfully treated with no or few anticancer drugs.

**Summary:**

The biggest weakness of current cancer treatments is that they only treat the cancer and not the actual patient. The “alkalization therapy” that we advocate does not compete with any of the current standard treatments, but improves the effectiveness of standard treatments, reduces side effects, and lowers medical costs.

## Introduction

Why did we decide to work in this kind of therapy? An experience was the beginning of this work. About 20 years ago (2001), when I was working at a university hospital, a patient with inoperable adenocarcinoma of the lung, clinical stage 3B, came to see me three years later. I had told him that his prognosis was probably about six months, but three years later he came to see me in good health.

When I asked him what he did to overcome the advanced cancer, he told me that he changed his diet. The diet was a low-calorie diet consisting mainly of vegetables and brown rice. I realized at the time, ‘If you don’t change the body of the cancer carrier, the cancer will not become suppressed. This can only be achieved through diet. It can only be reached by diet.

In the books I read later titled ‘Radical Remission’, the author, Kelly Turner, describes that “The first of the nine things that people who have had radical remission in common is changing their diet and drinking water.” (1).

## Objectives of the Study

Our aim was to consider the mechanisms by which cancer develops and to explain how these mechanisms can be applied clinically. Is it possible to stop the progression of cancer by changing the body of a person who has cancer? If the cancer stops growing, what is the mechanism by which this happens? Can it be cured? These are the questions that our research aims to answer.

## Theoretical Basis of Alkalization Therapy

When we think about how it is that cancer can form in the human body, there must be some mechanism at work. An interesting experiment to consider this was reported 70 years ago. Fibroblasts of cardiac tissue are cultured under atmospheric conditions; they can be normally passaged. The fibroblast cultures were injected with nitrogen instead of air in a sealed container, and when about half of the cells had died, they were returned to their original atmospheric conditions. As a result, cancer cells developed in the nitrogen-injected culture, but no carcinogenesis was observed in the atmospheric conditions (2). It was during this period that Warburg studied the metabolism of cancer and discovered its essential nature. He published a review in Science in 1956 with the title ‘On the origin of cancer cells’ (3). In his review, Warburg described the work described above. The implication of this experiment is that the presence of nutrients but the absence of oxygen causes cancer. What happens when there is no oxygen? Mitochondria breakdown occurs in the cell. When the mitochondria break down, cytochrome C is released from mitochondria and the cell undergoes apoptosis and dies. Warburg et al. observed that in cells maintained under such anaerobic conditions, glycolysis was enhanced, and oxidative phosphorylation was reduced. This is what Warburg calls a cell with enhanced fermentation and reduced respiration. In other words, under anaerobic conditions, cells that avoid mitochondrial breakdown and choose to live by glycolysis are ‘cancers’. This activated aerobic glycolysis, which is called the ‘Warburg effect’ is a hallmark of cancer metabolism and is known to be common to all cancers (4, 5).

It is a common understanding in biology that we, eukaryotic multicellular organisms, are made up of ‘eukaryotic cells with the characteristics of archaea and the mitochondria formed by α-proteobacteria’ (6, 7), and cancer cells are ancestral to archaea in terms of energy metabolism. In most cancer cells, mitochondria do not use oxygen, but do only substrate-levels phosphorylation dose produce minimal energy to maintain their own mitochondrial membrane potential. Cells that are forced to live on glycolysis in this way gradually develop abnormalities in gene expression. In most cases, this dysregulation occurs epigenetically in most cancers, but in rare cases, mitochondrial mutations have been reported to cause cancer recently.

More recently, Seyfried has proposed that cancer is not caused by a genetic abnormality but that it is a mitochondrial metabolic disease. He has experimentally shown that the cause of cancer is in the cytoplasm of the tumor, not in the nucleus. This is also the same proposal made by Warburg. The authors agree with this view and the importance of treating cancer as a metabolic disease (8, 9). Moreover, cancer patients with type II diabetes are known to have a poor prognosis (10), but we use metformin as needed to keep their hemoglobin A1c levels below 6, preferably around 5.8. When cancer cells start to live on glycolysis, their intracellular pH is always alkaline. In contrast, the intracellular pH of normal cells is almost neutral (11). Since cancer cells that live by glycolysis generate large amounts of H+ protons, they activate a mechanism to expel protons from the cell to keep the intracellular pH alkaline (12). As a result, the tumor microenvironment (TME) becomes acidic. Furthermore, extracellular acidic pH and intracellular alkaline pH of cancer cells is known to induce malignant behaviors, such as increased invasion and metastasis, multi drug resistance, and suppression of immune surveillance (13). One interesting example of this was reported in human lung cancer cell culture experiments, where 0.4 increase in intracellular pH was associated with a 2000-fold increase in the level of doxorubicin resistance in the tumor (14), proliferation and metastasis, expression of genetic abnormalities, growth factor activation, MDR and multidrug resistance, and vascular proliferation are activated.

It is well known that current cancer treatments often leave the TME acidic, resulting in poor therapeutic efficacy and severe side effects (4, 15). Therefore, our alkalization therapy aims to change the acidic TME to an alkaline. The actual methods are dietary interventions and the oral or intravenous administration of drugs that alkalize the body. As our bodies are made from the food that we eat, we believe that the act of alkalizing the body via food is a logical approach. A method to measure the pH of the TME has not yet been established to date, and hence we use urine pH as a surrogate indicator. The reason for this is that through our clinical practice, we have experienced that the urine of most patients who have achieved radical remission has an alkaline pH of 7.5 to 8. In the literature, it has been reported that Na+-H+ exchange 1 (NHE-1) becomes inactive when the extracellular environment is alkalized. NHE-1 has high ion transport activity under acidic conditions and its activity decreases with the shift to alkaline conditions, and that its activity decreases with the shift to alkalinity and completely disappears around pH 7.5 (16, 17). Thus, whether alkalization of TME really improves the therapeutic effect in clinical practice is an issue that should be examined in the future.

## Limitations of Deductive Method and Inductive Approaches: Learning From Hegel’s “Dialectic”; Paradigm Shift From EBМ To SBМ

Georg Wilhelm Friedrich Hegel was a famous philosopher who advocated dialectics. Today’s medicine is based on the concept of Evidence Based Medicine (EBM). This EBM is a “deductive method”. What would you do if you were a cancer patient and you were told that stage 4 cancer cannot be cured, that there is only treatment to suppress it, or that treatment will not cure it? In deductive reasoning, we start from a given hypothesis and predict ‘what we will see’ if the hypothesis is true. In deductive reasoning, this prediction of ‘what we will see’ is always objective in the sense that it will be true if the hypothesis is true. The problem is that we cannot expand our knowledge beyond what is in the hypothesis. The biggest problem is that current medicine treats stage 4 cancer based on the assumption that it cannot be cured. To solve this problem, it is important to study people who have been cured of stage 4 cancer. In other words, it is important to “try to evaluate the significance of the results observed in a single experiment,” i.e., to find “inductive” evidence. This combination of inductive and deductive perspectives provides a purely deductive method (called objective probability calculation) for drawing scientific conclusions from inductive ends (18).

From the point of view of Hegel’s dialectic, the current EBM includes the antithesis that ‘it is not possible to cure stage 4 cancer while receiving anticancer drugs. As a proposition to deny this, it is important to construct a hypothesis because ‘there are people who have been cured of stage 4 cancer’, and to perform inductively what those who have been cured have done in the treatment of stage 4 cancer. At present, it appears most appropriate to construct the hypothesis based on current molecular biological knowledge. Based on the results (Aufheben), the subsequent hypothesis needs to be tested, and new EBM needs to be constructed. Medical treatments based on the molecular biological findings necessary to construct this hypothesis is what is referred to as science-based medicine (SBM).

## How Do We Deal With Cancer?

### Alkalinizing the Acidic Tumor Microenvironment

So, what should we doctors do in actual clinical practice? As we have already mentioned, cancer is a cell that is based on a metabolic disorder, so the first thing to do is to stop this disorder. Cancer is said to undergo ‘selfish’ metabolic reprogramming, and it was mentioned above that the pH, which indicates the acid-alkaline level inside the cancer cell, must become ‘alkaline’. To maintain the homeostasis of intracellular alkalization, ion transporters, such as NHE-1, vacuolar H+-ATPases, monocarboxylate transporters and carbonic anhydrases are expressed on the surface of cancer cells (12). The first thing to do is to stop these proton transporters.

**Figure 1:**
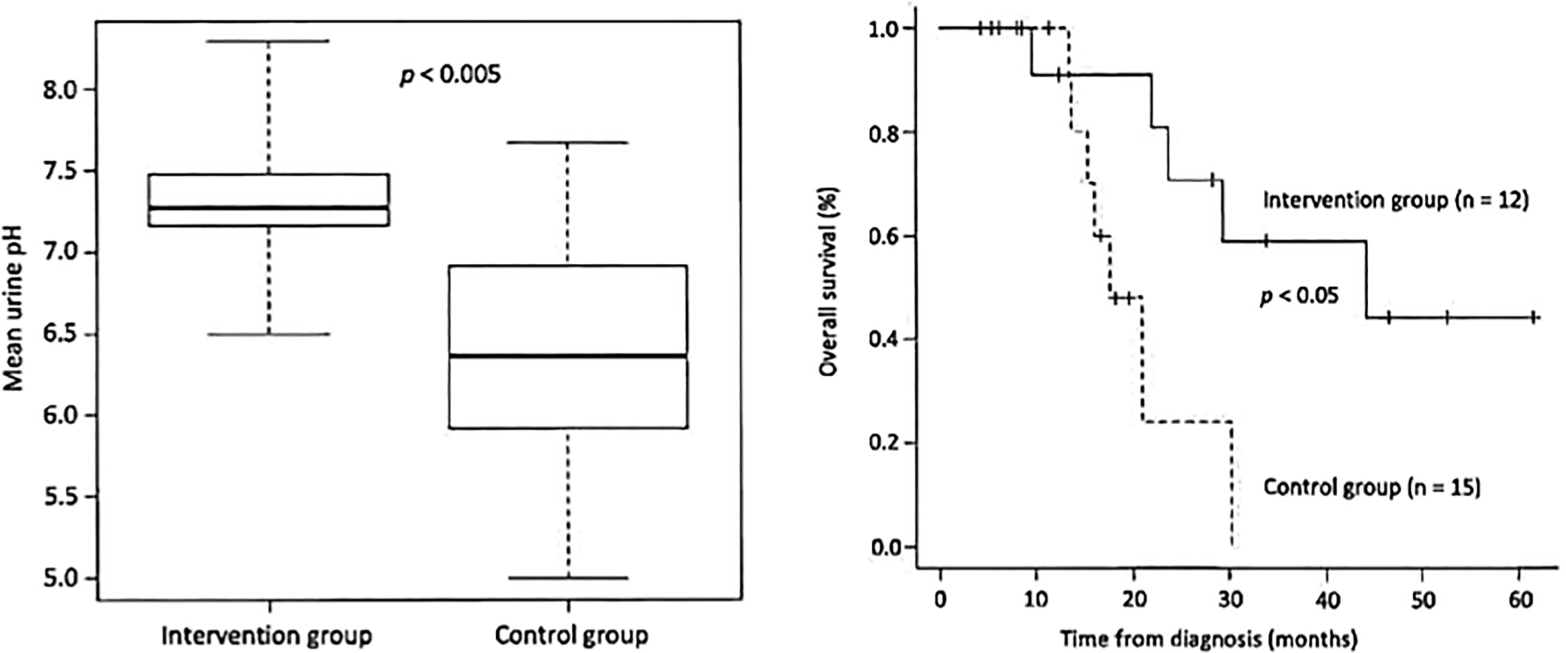
(Left) The mean urine pH of the alkalization group of small cell lung cancer patients is shown to be higher than that of the control group. (Right) The overall survival of the alkalization group of small cell lung cancer patients is shown to be prolonged compared with that of the control group.

The patient should first be advised to change his/her diet. It is important to measure the patient’s urine daily with a litmus test paper. Under normal conditions, the urine pH is almost 5-5.5 acidic, but as the diet is changed to an alkalinizing diet (fruits and vegetables, no meat, no dairy products), the patient’s urine pH will increase. The aim is to achieve a urine pH of 7.5-8 or higher, and if this is not achieved, alkalinizing agents may be given intravenously or orally. **Figure 1** is from our paper published in a journal and shows that in cases of small cell lung cancer, the urine pH was approximately 7.3 in the alkalization group and 6.4 in the control group, indicating an increase in urine pH in the alkalization group that showed efficacy increase in the alkalization group (19). Several other reports have also been made (20, 21).

The next thing to do is to reduce inflammation. C-reactive protein (CRP) is used as an indicator. The patient should be taught to keep the CRP below 0.05. It is reasonable to assume that the elevated CRP in carcinoma carriers is due to acidification of the TME, where protons present in the TME attract primary immune cells such as neutrophils and macrophages (22, 23). A rapid reduction in CRP can be achieved by ‘donating’ electrons to the patient. A large intravenous dose of ascorbic acid (at least 4 grams) is very effective. Warming the patient’s body and making him sweat are also helpful. Obesity also raises CRP, so patients should be advised to reduce their weight. At this time, it is important to improve the bacterial flora in the intestine. There is a growing consensus that abnormalities in the intestinal microflora are involved in carcinogenesis [Schwabe & Jobin (24) Zambirinis et al. (25) Johnson et. al. (26) Yu & Schwabe (27) Gopalakrishnan et al. (28) Routy et al., (29) Riquelme et al., (30)]. It has been reported that the human gut microbiota regulates many host processes, including metabolism, inflammation, immunity, and cellular responses, and that its composition is known to be altered in many diseases, including cancer (31). The gut microbiome may also affect the development of cancer. It has also been reported that the microbiome can worsen the prognosis of cancer, by producing carcinogenic toxins and metabolites. Therefore, to improve the gut microbiome, foods rich in ‘water-soluble pectin’ should be consumed.

The next step is to ‘suppress the primary immunity and activate the secondary immunity’ (32). Cancer is known to utilize inflammatory cytokines to grow. In the absence of cancer, this response is the first response in the “wound-healing process” and is called the primary (innate) immune response, but it is also known to support cancer growth (22). Normally, TMEs are acidic. Recently, it has been known that this acidification has a beneficial effect on cancer cell proliferation, migration, invasion, metastasis, therapeutic response and the function of stromal cells such as immune cells and vascular cells in cancer growth. It is believed that the activation of proton-sensitive GPCRs by acidosis causes the above conditions. Considering this aspect, it is safe to assume that elimination of protons presents in TME, i.e., alkalization of it, can inhibit cancer growth. Acidification of the TME is associated with the accumulation of myeloid-derived suppressor cells (MDSCs), which are a population of cells that proliferate during cancer, inflammation, and infection, and have a robust ability to suppress effector T-cell responses (33). It has been reported that about 80% of MDSCs eventually differentiate into neutrophils and the remaining 20% into macrophages in the TME, and that alkalization of the TME prevents these MDSCs from accumulation in the TME. When the TME becomes alkaline, the primary (innate) immune response is suppressed, but as a result the secondary (acquired) immune response becomes more active. The white blood cells that play a central role in this process are lymphocytes. There are several subsets of lymphocytes but identifying the specific subset does not appear to be important in making clinical decisions.

In our clinical experience, we found that patients who show improvement have a neutrophil/lymphocyte ratio of less than 2, or even less than 1.5, and a lymphocyte count of more than 1,500, or even more than 2,000, which we use to determine the clinical status of the patient. CRP is also useful in determining neutrophil activity, and a CRP of 0.05 or less is considered as low neutrophil activity. The goal is to achieve a urine pH of 7.5 to 8.0 or more. Once this is achieved, it is often our experience that anticancer drugs can be given at less than half the standard dose and still show satisfactory effects.

### Causes of Drug Resistance, Invasion, and Metastasis of Cancer

It is well-known that cancer cells that survive anticancer drug treatments gradually become resistant to the drugs. This is closely associated with acidification of the TME around a cancer. Alkalization of the TME around malignant cells is known to attenuate the intracellular concentrations of anticancer drugs, such as vinblastine, adriamycin, cisplatin, paclitaxel, campthothecin, etc. (4), and hence tumors become resistant to anticancer drugs when the TME becomes acidic (34). As mentioned above, Keizer and Joenje H (14) demonstrated using human lung cancer cells that an increase in intracellular pH from 7 to 7.4 results in very high drug resistance, such as a 2,000-fold increase in adriamycin resistance. There is a strong positive correlation between the degree of MDR and the intracellular pH of cancer cells. This mechanism is likely to be associated with the high acidification of intracellular organelles (lysosomes, endosomes, Golgi network, etc.). The so-called protonation, ion capture effect, or the theory of ‘Mathematical Modelling of Tumor Acidity Regulation of Intracellular pH’ has been proposed to explain this hypothesis (35). In addition, with regard to metastasis, NHE-1 also regulates the formation of invadopodia (cellular structures that mediate the migration and invasion of tumor cells). Therefore, reducing NHE1 activity in cancer cells is linked to the inhibition of metastasis, and this can be achieved by ‘alkalization’ of the TME (11). In conclusion, tumors acquire resistance to anticancer drugs and the ability to metastasize because of the acidification of the microenvironment surrounding the tumor (TME), and therefore, alkalization of the TME is expected to substantially reduce cancer activity.

### Purification of the Arterioles

Carcinogenesis occurs when, as Warburg states, ‘nutrients are supplied but oxygen is lacking’. It is reasonable to assume that this is true in the human body because of the narrowing of the arterioles. When the narrowing of the arterioles occurs, it becomes difficult for blood cell components to flow, but liquid components can flow. The flow of fluid means that nourishment is being carried out, and the difficulty in the flow of blood cells means that without the flow of red blood cells, it is difficult for oxygen to be supplied. When white blood cells become blocked, a cytokine storm develops, leading to chronic inflammation (36). Most cancer patients consume large amounts of high-fat food. In addition to eating meat and drinking heavily, many male patients also smoke. Many female patients prefer sweet cakes with lots of cheese and cream. These dietary habits may be considered as a cause of narrowing of the arterioles.

In addition, soft stools and diarrhea are more common in male patients and constipation is more common in female patients. These are all associated with dysbiosis of the intestinal microflora. The gut microbiota as above also needs to be purified to become synbiotic (37). This study by Zitvogel showed that alterations in the human gut microbiota can lead to cancer. By avoiding high-fat diets and purifying the gut microbiota to a symbiotic state, it is possible to stop cancer in its tracks.

### Anti-Tumor Effect of Intravenously Administered Ascorbic Acid

In our clinic we often use intravenous ascorbic acid in the treatment of cancer. There has been much debate as to whether ascorbic acid (vitamin C) is effective in the treatment of cancer, but it has recently been shown that the pharmacological effects of taking this substance orally and administering it intravenously are quite different. Although the function of vitamin C in vivo is not clearly and fully understood, it is understood that this substance acts in vivo as an electron donor (38). The antitumor effect of ascorbic acid can only be achieved by intravenous or intrathoracic or intraperitoneal administration. Oral administration does not have such an antitumor effect, which the authors intend to report in detail elsewhere (39–41).

## Discussion

The main problem with current cancer treatment is that it does not consider the condition of the cancer patient’s own body. As stated earlier, “In conclusion, cancer is the result of cells being forced to choose glycolysis as a metabolic pathway to survive in the presence of nutrients but without oxygen. In the case of cancer, it is reasonable to assume that the first priority is to restore the body of the person with cancer to the state it was in when there was no cancer. In other words, it is important to ‘make the cancer patient’s body less susceptible to cancer’ before treating it. All multicellular organisms are made up of eukaryotic (nucleated) cells. Eukaryotic cells emerged from prokaryotes 4 billion years ago through mitochondrial symbiosis, resulting in a drastic expansion of the number of genes expressed by 200,000 (6–7). This leap in genomic capacity, due to the power of mitochondria, was a prerequisite for the key evolutionary processes that led to the increasing complexity of eukaryotes (multicellular organisms) (6). Based on this complex network of genes and considering the results of the experiments of Goldblatt & Cameron (2), we can understand that hypoxia induces induction at the genetic level, the accumulation of which results in cancer.

Otto Warburg, studying cancer metabolism, states that ‘the characteristics of cells which have survived hypoxia are altered, there is an increase in fermentation (glycolysis) and a decrease in respiration (oxidative phosphorylation), and these characteristics accumulate’. This first event in carcinogenesis has recently been reported to be the transmission of signals from the mitochondria to the nucleus, resulting in the condition described above. It is reasonable to assume that avoiding ‘mitochondrial break-down’ is a survival strategy for cells in oxygen-deprived conditions, as it does not lead to cell death. Mitochondrial retrograde signaling is a mitochondrial-to-nuclear signaling pathway that influences many cellular and biological activities during normal and pathophysiological conditions. In yeast, it is used as a sensor of mitochondrial abnormalities and initiates the readjustment of sugar and nitrogen metabolism, for which the RTG gene has been found to be responsible (42–45). However, although a similar response has been observed in human cells, the signaling players remain unclear.

Retrograde signaling has been suggested to be involved in tumor progression by inducing the invasion of non-neoplastic cells. It has been speculated that Nuclear Factor κ B (NFκB) may be the master regulator of this network in humans (46). In most tumors, the Warburg effect is a “selfish” metabolic reprogramming, with an initial overexpression of hypoxia-inducible factor-1 (HIF-1) (15). This suggests that the human retrograde signaling pathway is based on the activation of NFκB in the absence of oxygen, which somehow transmits a signal from the mitochondria to the nucleus to avoid mitochondrial disruption, the first genetic abnormality being the overexpression of hypoxia-inducible factor-1 (HIF-1). When carcinogenesis is triggered, the surrounding environment of the cancer cell becomes acidic and the cell adapts to this, leading to malignant transformation of the cell (47). When this occurs, the pH within the cancer cell becomes alkaline and the TME becomes acidic. The regulation of this intracellular alkalization and acidification of the extracellular microenvironment is crucial in the treatment of cancer.

There are many studies on proton pump inhibitors (PPIs) as a drug repositioning therapy targeting acidic TME (48–51). Several in vivo and in vitro studies have shown that combination of PPI and chemotherapies increases the chemotherapeutic responses (52–54). Furthermore, preclinical studies in human tumor cell lines have reported that administration of PPI alone, without chemotherapy, induced apoptosis of cancer cells and produced anticancer effects (55–57). Population-based studies have also suggested that PPI use may prevent the development of breast cancer (58–60). Although clinical studies are limited, three patients of advanced colorectal carcinoma were treated with high-dose PPI in combination with chemotherapy and reported favorable results (61). Furthermore, for patients with metastatic breast cancer, the combination of chemotherapy and PPI was reported to significantly prolong time to progression and overall survival compared to chemotherapy alone (62). On the other hand, several animal studies have also shown that systemic alkalization with buffer therapy using bicarbonate and/or alkalizing agents inhibits tumor progression (63–65). Moreover, although Amiloride and Cariporide are known as drugs that stop alkalization in cancer cells, they have not yet been applied clinically due to their adverse effects. There are many reported intervention methods to neutralize acidic TME, most of which are based on the concept of proton control by external intervention of drugs. ‘Alkalization therapy’ we advocate alkalizes TME using an alkalinizing diet and alkalizing agents to cleanse the body of cancer patients.

Alkalinizing diet and alkalinizing agents are used in the treatment. However, the measurement of the acid-alkaline level of the microenvironment surrounding the cancer is not available in current clinical practice (12, 66, 67). We found that in all cases where various cancer treatments were effective using our ‘alkalization therapy’, the urine pH became alkaline (19–21, 68, 69). Due to the impossibility of measuring the pH of the microenvironment surrounding the cancer, we use the patient’s urine pH as a surrogate indicator to practice alkalization of it. This alkalization of the urine pH means a decrease in the number of acidic substances such as protons excreted in the urine and does not imply an extreme alkalization of the acid-alkaline balance of the body fluids.

When the TME is acidic, cancers are more resistant to all therapies, including anticancer drugs, radiotherapy, immune checkpoint inhibitors and molecular targeted therapy, and the side effects of these therapies are more severe (4, 47, 70). In the treatment of cancer, it is important to suppress primary immunity and activate secondary immunity. The primary immune system began over hundreds of millions of years ago when a series of specialized enzymes and proteins evolved to protect our primitive ancestors from attack by the outside world. This inflammatory immune response worked so well that its function has been conserved. Until now, cancer researchers have treated cancer with the belief that genes are the main underlying cause of cancer. However, in the last few decades we have come to understand that the inflammatory component of the primary (innate) immune system, which is normally part of the wound healing process, promotes carcinogenesis by aiding tumor development and growth. The reason why the inflammatory component of the primary (innate) immune system is activated in cancer development and growth is due to the acidification of the TME caused by the extracellular excretion of intracellular protons from glycolysis. It has been reported that cancer cells are more active in this acidic environment (22, 71). The tumor microenvironment is acidic. This acidification has a multifaceted effect on cancer cell proliferation, migration, invasion, metastasis, resistance to treatment and the function of stromal cells such as immune cells and vascular cells. However, the molecular mechanisms by which cancer cells and stromal cells sense and respond to acidic pH in the tumor microenvironment are not well understood.

The role of the family of pH-sensing G protein-coupled receptors (GPCRs) in tumor biology is increasingly being recognized as important to regulate cancer cell metastasis and proliferation, immune cell function, inflammation, and angiogenesis. Neutrophils sense protons of the TME via GPCRs expressed on their cell surface and aggregate in areas of high proton concentrations. Macrophages accumulate by a similar mechanism (72, 73). Acidification of TME is associated with pro-tumor polarization of tumor-associated macrophage phenotype (74), and it is reported that Inhibition of tumor acidosis through PPIs promote an antitumor phenotype of tumor-associated macrophages (75). MDSCs are a population of cells that proliferate during cancer, inflammation, and infection, and have the robust ability to suppress T-cell responses (33, 76). MDSCs accumulate at sites of activation of the innate immune system by the activity of neutrophils and macrophages, as described earlier, and NO and ROS are said to be involved in this mechanism. About 80% and 20% of MDSCs differentiate into neutrophils and macrophages, respectively, and if the acidic TME is left without intervention, inflammation in this environment will increase. It hence appears reasonable to assume that acidification of the TME is the most common cause of the resistance of cancers to treatment. Our strategy of alkalinizing the acidic TME is called ‘alkalization therapy’.

The aims of this treatment are to achieve (1) a urine pH of 7.5 to 8.0 or more, (2) a CRP level of 0.05 or less, and (3) a neutrophil/lymphocyte ratio of 2.0 or less (1.5 if possible), and a lymphocyte count of 1,500 to 2,000 or more. The cause of the increased CRP is thought to be the presence of H+ and the development of arteriolitis owing to vascular narrowing in hyperlipidemia, obesity, diabetes, and heavy drinking. Alkalization of the body is very much influenced by the diet consumed and is determined using urine pH. In addition, the association between intestinal microflora and cancer will require further study in the future.

## Conclusion

The treatments we advocate do not in conflict with conventional standard therapies but can be used in combination to increase their efficacy and reduce side effects. The human body is a dissipative structure, which means that it is an open system that is not in thermodynamic equilibrium. This means that it is possible to reduce the entropy of the body. It is our experience that alkalization of the TME enables various treatments to become more effective, and with the future collaboration of many researchers, we hope that it will soon become possible to treat intractable cancers using standard treatments combined with alkalization therapy (19, 21, 21, 68, 69, 77, 78).

## Patients Demonstrating a Successful Treatment Response to Alkalization Therapy

The cases shown here are patients whose treatment involved no anticancer drugs or, if used, exceedingly small amounts of oral medication. These case reports are comprehensively included in “Investigation of survival factors for cancer patients using data science methods” approved by Institutional Review Board of the Japan-Multinational Trial Organization. Written informed consent for publication of these case reports and accompanying images has been obtained from the patients.

(i) Male, 84 years old (at the time of first consultation), Unresectable renal pelvis cancer, cT3N2M0, Periaortic lymph node (LN) metastasis (+)

First visit to our hospital February 2021, January 2021: Visited T Hospital due to gross hematuria; CT scan showed venous invasion of right renal pelvis carcinoma and pararenal aortic LN metastasis. The same diagnosis was made at the University Hospital. He was told by the doctor that anticancer drug treatment would not be given because he was too old, and it would not be effective. An alkalizing diet and intravenous vitamin C showed a marked improvement in the renal pelvis cancer. No anticancer drugs were used.

**Figure 2:**
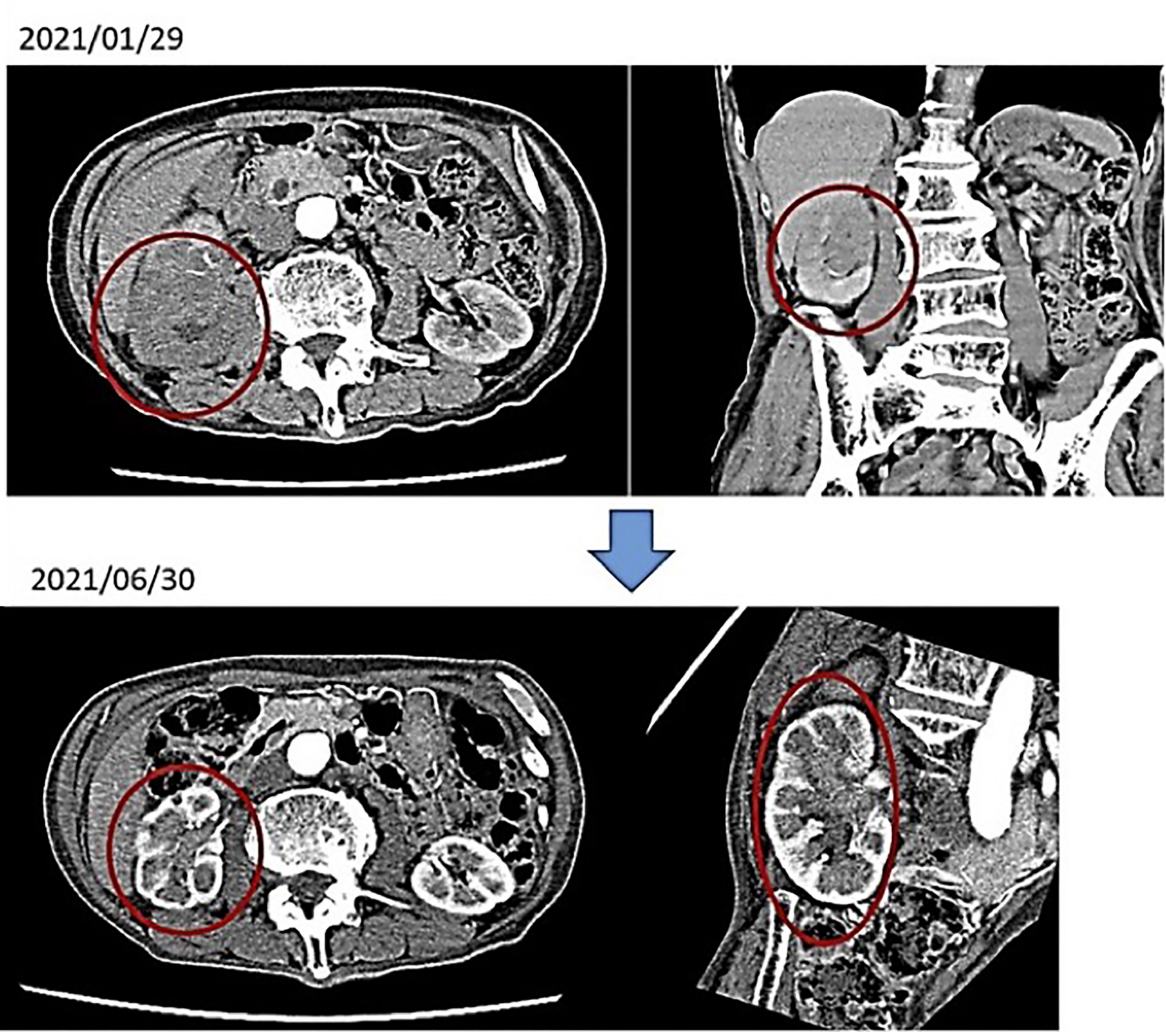
A contrast enhanced computed tomography of abdomen are shown.

**Figure 3:**
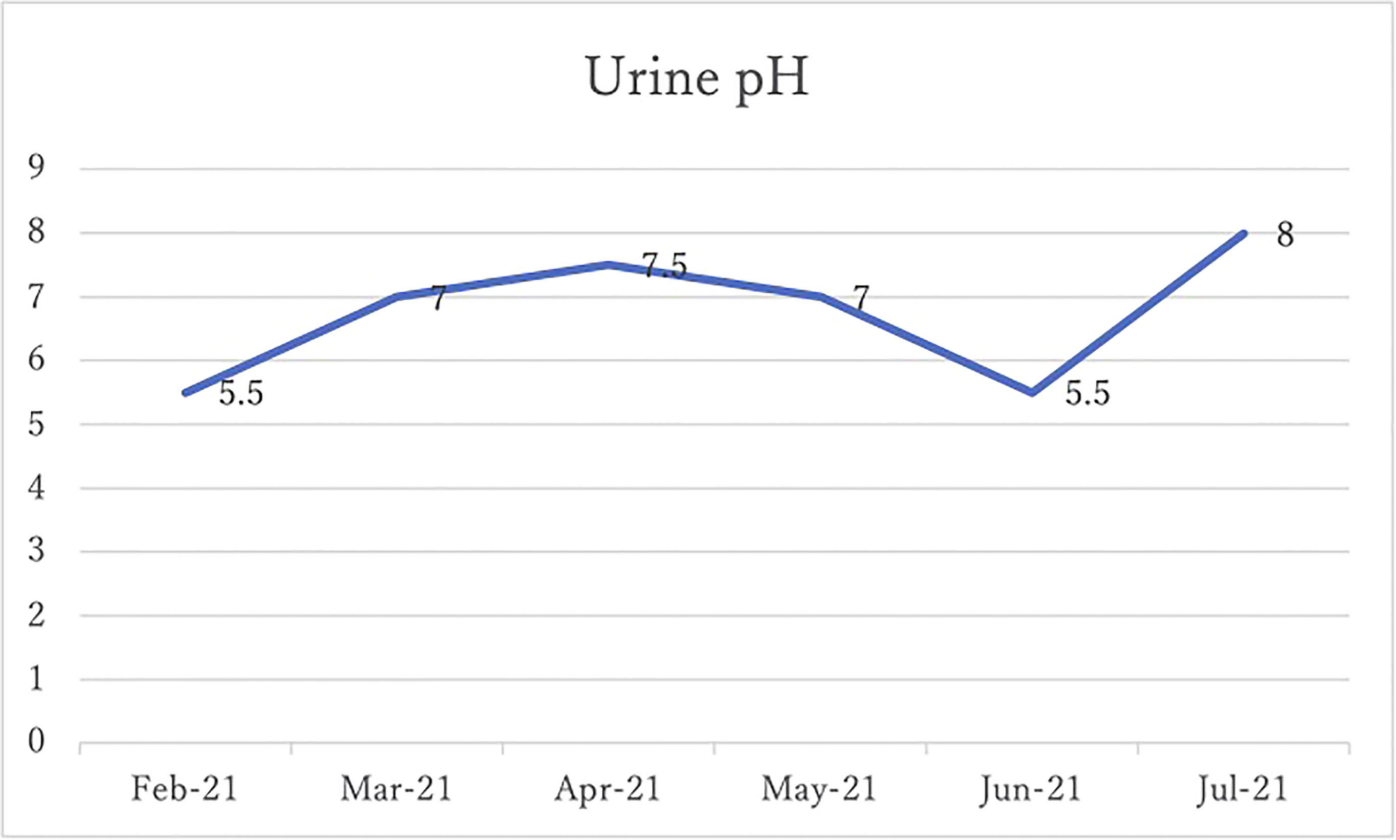
His urine pH levels are shown.

After 4 months of alkalinizing diet + alkalinizing agents + intravenous vitamin C, the tumor in renal pelvis of the right kidney, which was found in January 2021, was significantly reduced in June 2021. The red circles indicate the sites of renal pelvis cancer (**Figure 2**). In February 2021, urine pH = 5.5, which increased to 8 in July (5 months after the visit) (**Figure 3**).

(ii) Female, 76 years old, Malignant lymphoma of stomach, 2015/12, First visit to Wada Clinic.

**Figure 4:**
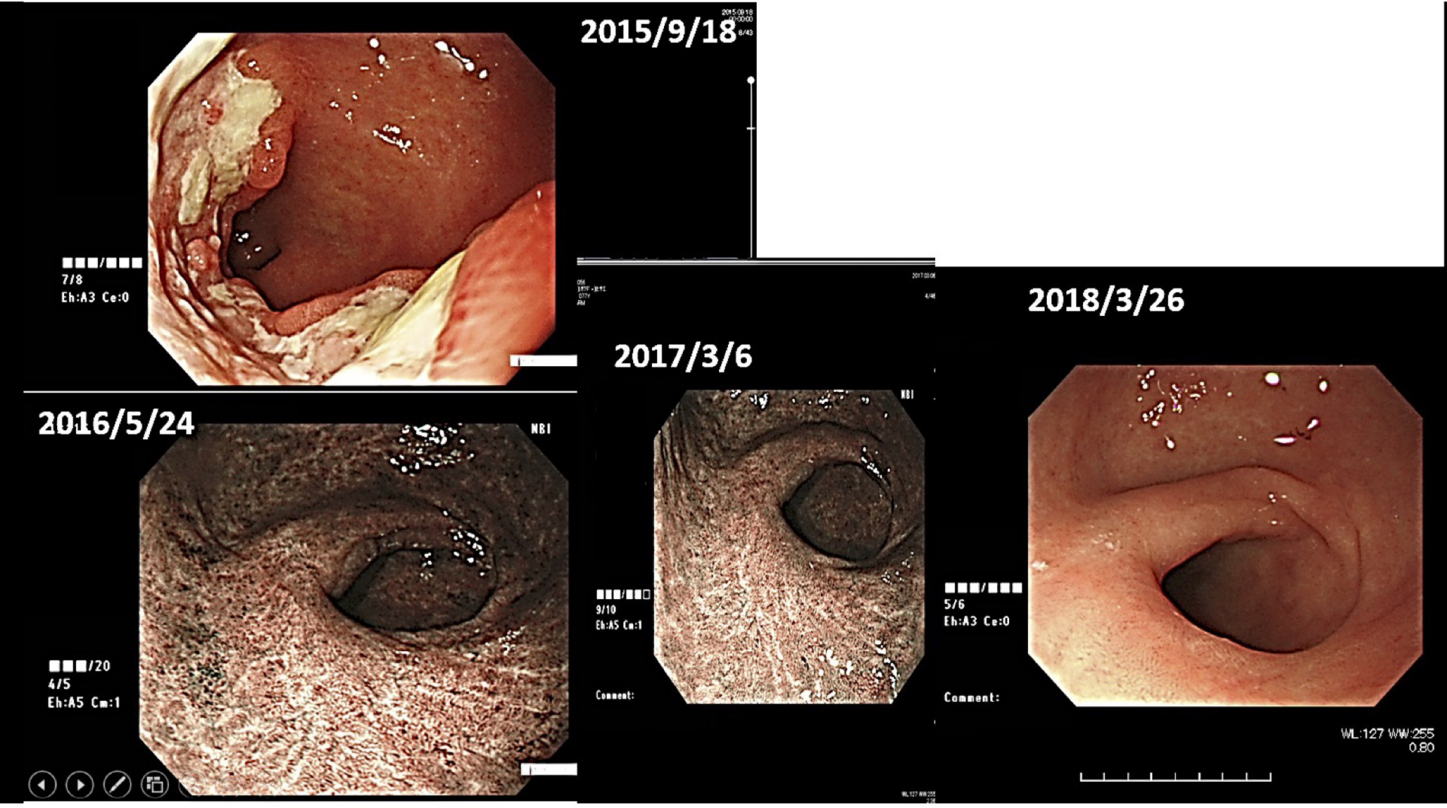
Her images of upper gastrointestinal endoscopy are shown.

Her gastric malignant lymphoma disappeared two and a half years after her first visit to Wada Clinic, simply by changing her diet (**Figure 4**). All she did was to start an alkaline diet as alkalization therapy.

(iii) Female, 89 years old, malignant lymphoma of the right tonsil.

In February 2009, the patient presented with an enlarged right tonsil and swollen lymph nodes in the right mandible, which were biopsied and a diagnosis of malignant lymphoma was made. The malignant lymphoma was diffuse large B-cell lymphoma, CD20 positive, CD79α positive and negative for epithelial marker A E1/AE3 negative.

The patient was advised to be hospitalized and receive anticancer therapy, and came to our clinic on April 2009 because of anxiety about side effects. At the time of the consultation, the patient had an ulcer on the right oral mucosa, which made opening the mouth a little difficult.

**Figure 5:**
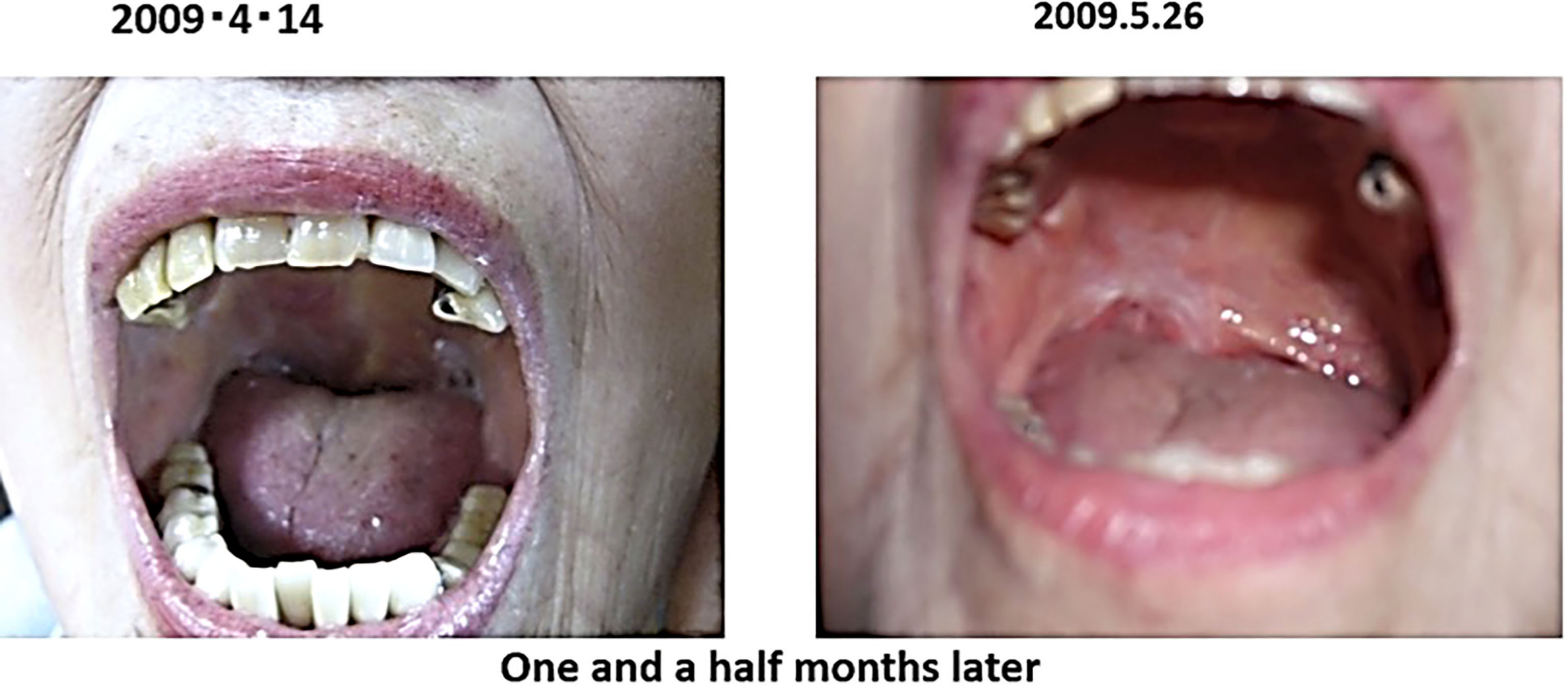
Her photographs of right tonsil are shown.

After the visit to our clinic, I recommended her to drink 2 bags of red bean cedar tea (Yunnan Yew tree) infused with 1 liter of tea per day to soak the affected area, and 3 bags of Misatol (plum extract supplement) in 3 portions to soak the affected area. The lymphoma disappeared after two months. The lymphoma almost disappeared after two months and has not recurred since (**Figure 5**). She lived her daily life in good health, but two years and eight months after the start of treatment, at the age of 91, she passed away as if asleep after breakfast one day.

(iv) Female, 15 years old, malignant lymphoma of tonsil.

**Figure 6:**
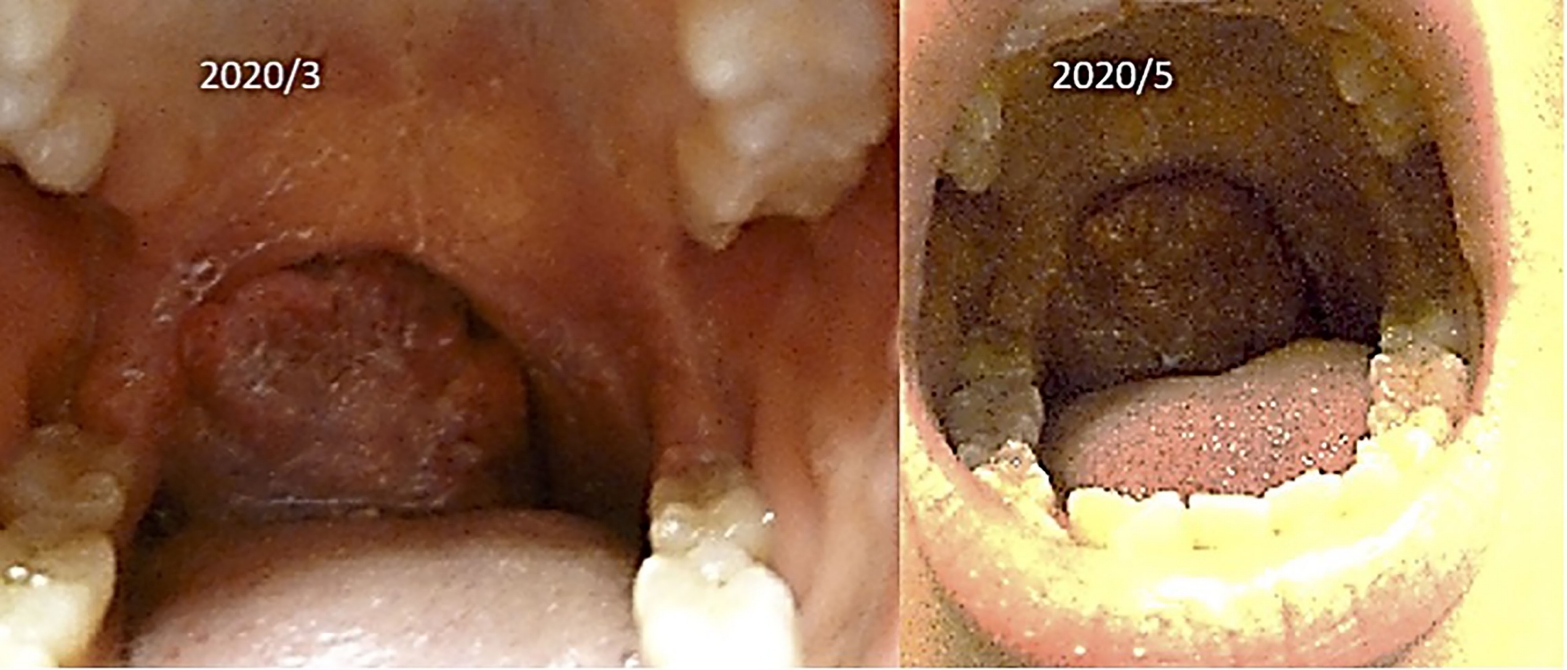
Her tumor of tonsil is shown.

**Figure 7:**
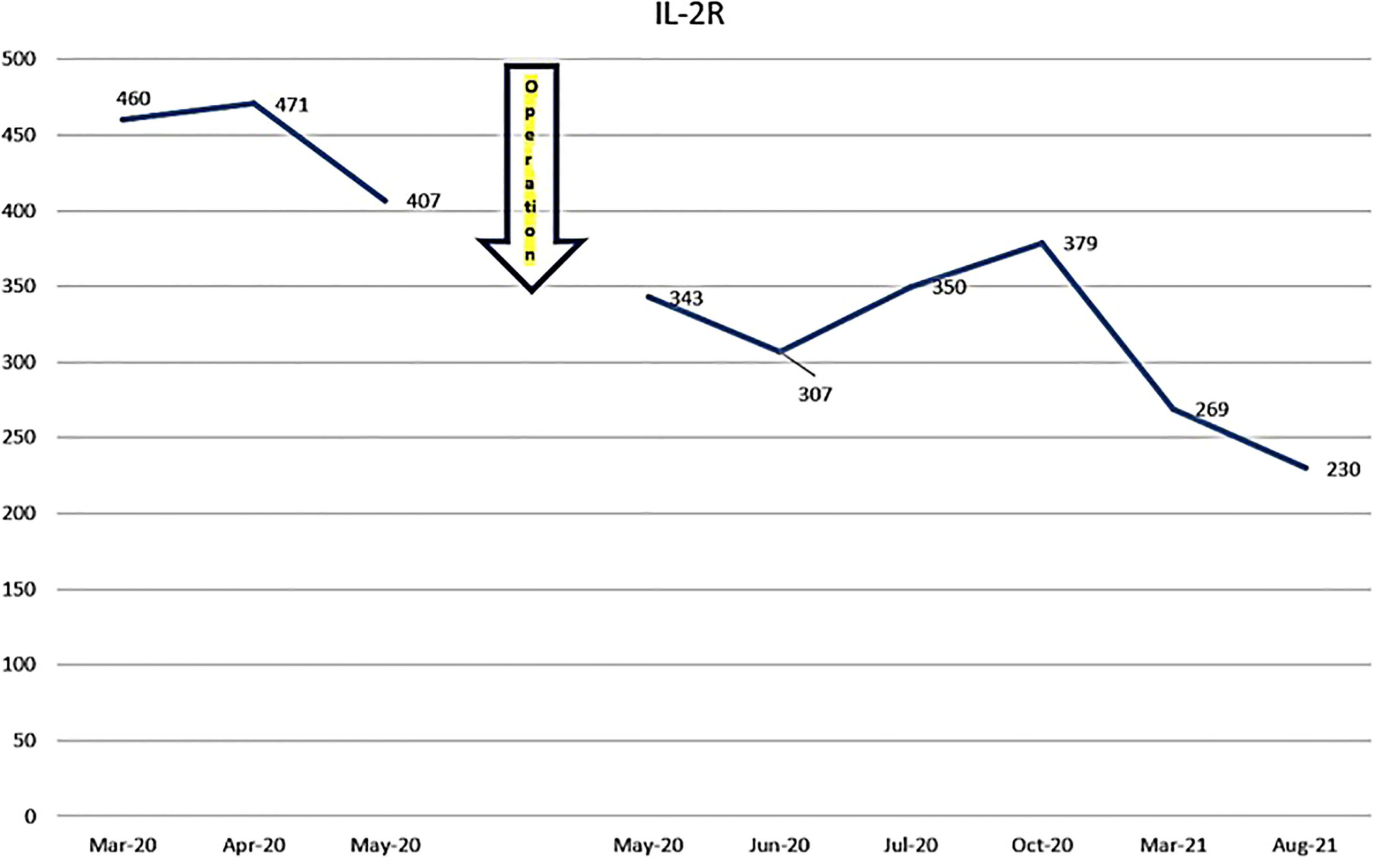
Her IL-2R levels are shown.

The patient was first seen at our clinic in 2020/3. She was diagnosed as diffuse Large B Cell Lymphoma (DLBCL) of tonsillar origin at the Children’s Hospital in 2019. PET-MRI showed no obvious accumulation except for enlarged right palatine tonsil. Prior to her illness, she was an imbalanced eater of foods. She was very fond of sweets. In the two months following the first visit, there was no increase in the lymphoma of the tonsils. However, in May 2020, the tumor became airway obstructed (**Figure 6**), causing breathing difficulties, and she underwent emergency surgery at the prefectural hospital. Since the resection, she has been taking apple pectin juice, citric acid preparations, red bean cedar tea (Yunnan Yew tree) and summer white chrysanthemum (feverfew) and has had no recurrence. She has not been treated with any anti-cancer drugs. Her IL-2R decreased after her visit to our clinic and has continued to decrease steadily after her surgery (**Figure 7**).

(v) Male, 64 years old. Gastric cancer (unresectable), multiple liver metastases, Adenocarcinoma(por), Group5. First visit to our clinic August 2020.

History of the disease: Introduced by Dr. B, Department of Surgery, Hospital A. On July, 2020, the patient was brought to the emergency room because of fatigue on standing up. When he came to the hospital, he was found to be anemic with Hb = 7.2 and was diagnosed with advanced gastric cancer on further examination. A contrast-enhanced CT scan revealed multiple liver metastases, and he and his wife decided not to take standard anti-cancer treatment and came to our clinic.

(Diet before the onset of the disease) Dairy products: a lot. 350ml of milk, a pack of yoghurt, a lot of cheese, taste: strong.

(Course) Recently, he has been cooking by himself. He eats an alkalizing diet with lots of vegetables and fruit. He wants to reflect on his diet and way of life and challenge himself to live with cancer. He doesn’t want to do standard therapy.

He has decided to do the following Things.

Start the Maruyama vaccine (immunostimulant) and receive lentinan (immunostimulants) intravenously once a week. Take one tablet of the oral anticancer drug TS-1 (5FU derivative) (20 mg) twice a week. Alkalization of the urine with citric acid preparations and sodium. The patient drinks 140 ml of apple pectin juice daily for three months and receives 25 g of Vit C intravenously. In order to increase the secondary immunity, mushrooms should be ground up and eaten in soups. Keep warm and exercise.

**Figure 8:**
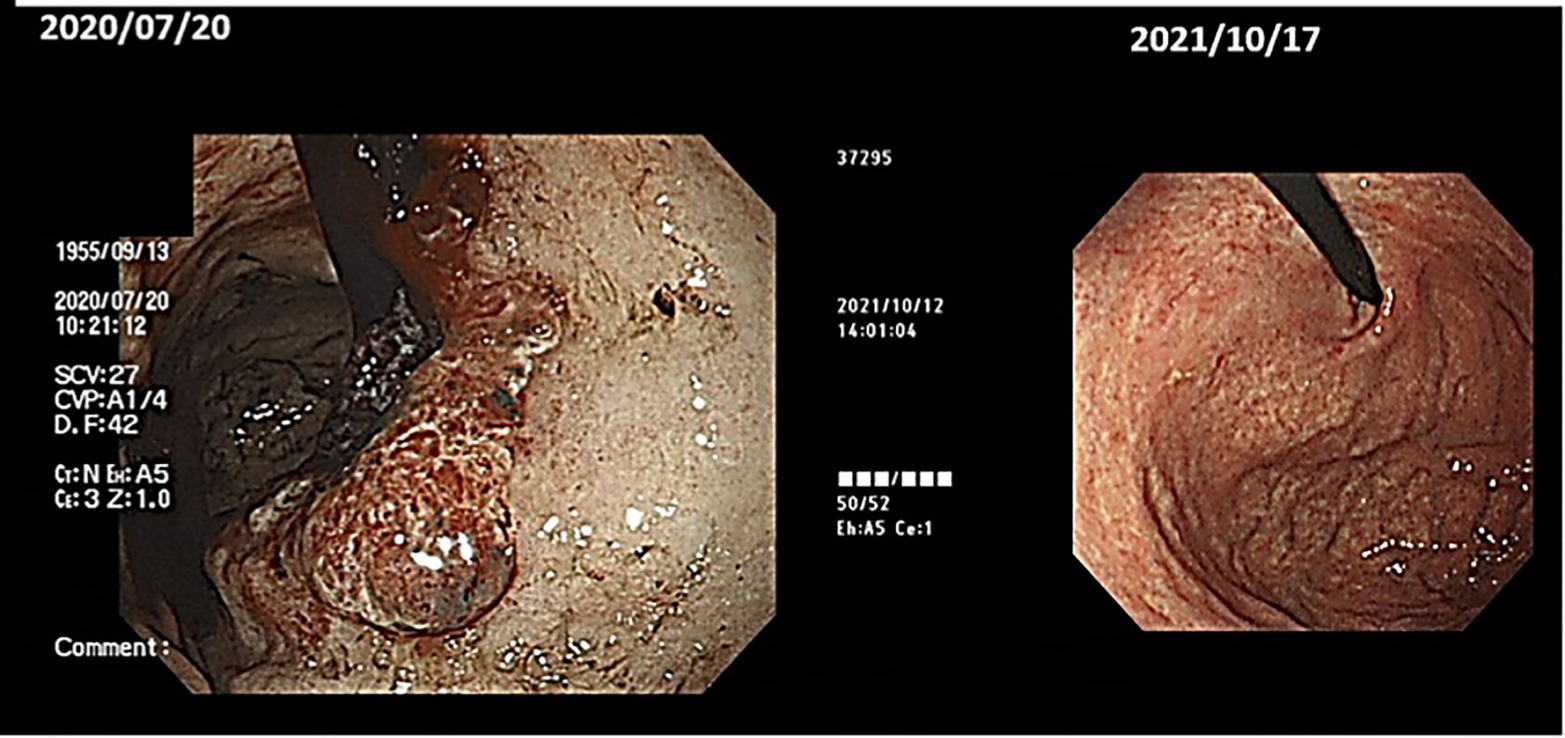
An upper gastrointestinal endoscopy showed that tumors had disappeared.

One year and three months later, gastric endoscopy showed that all cancers had disappeared (**Figure 8**), and PET scans also showed that all liver metastases had disappeared. (He has put this information on you-tube. The address is as follows: https://youtu.be/zHF_zUE9prI).

(vi) Male, 70 years old. Multiple gastric cancer, first visit in December 2012.

At the beginning of October 2012, he had a check-up at a hospital in Kagawa. He went to a hospital in December. He was told that he would have to undergo an immediate resection, but when he asked to wait until next year, he was told that the polyps in the upper part of the stomach would be removed under endoscopy, and that the lower part of the stomach would be removed and a subtotal resection would be performed.He was positive for H. pylori and was put on medication.

**Figure 9:**
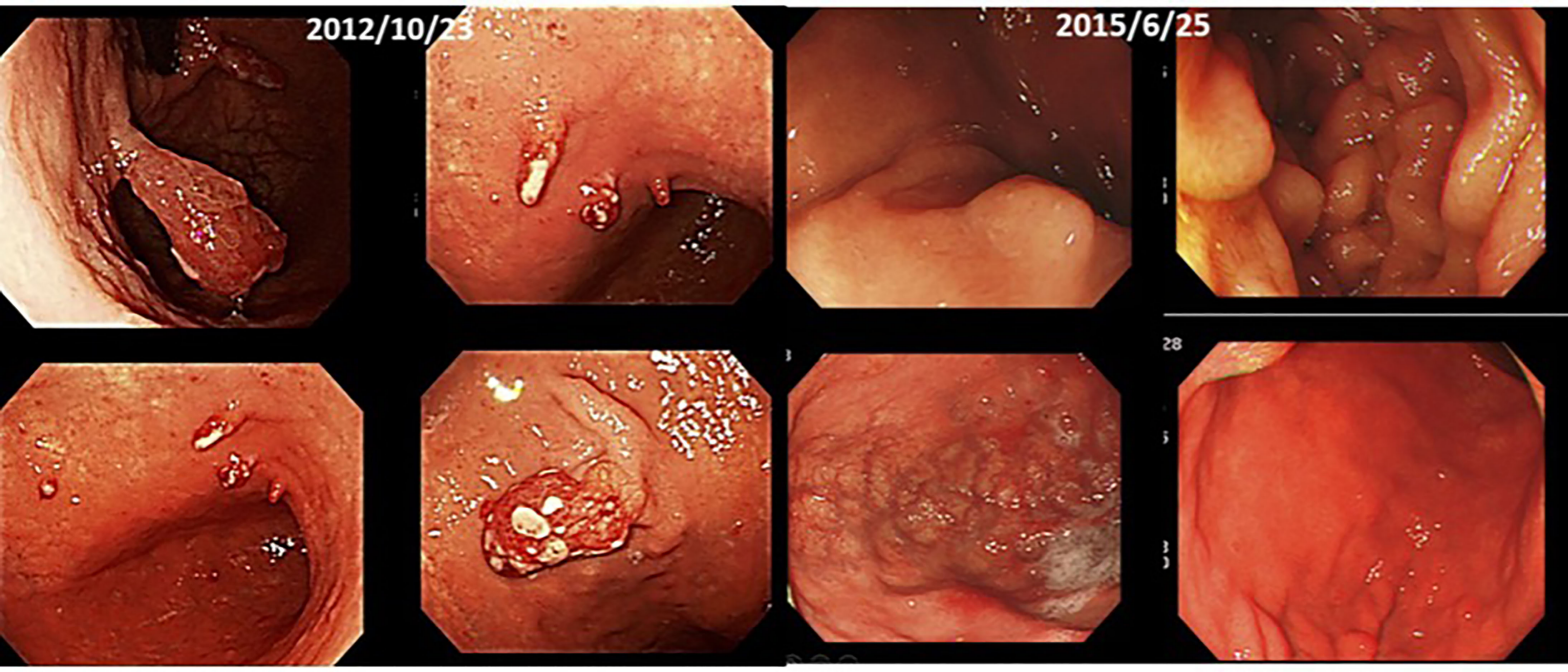
An upper gastrointestinal endoscopy showed that all tumors had disappeared.

He has been receiving intravenous lentinan (an immunostimulant) once a week since December 2012 and was started on TS-1 20 mg orally three times a week. After three years, his stomach cancer had disappeared after following a diet and lifestyle that was “light in taste, rich in fresh fruits and vegetables, dairy-free and meat-free” (**Figure 9**).

(vii) Male, 74 years old. First seen in our clinic on December 2014.

The patient was diagnosed with gastric cancer (early stage) after a thorough physical examination in 2012. He underwent gastrectomy (laparotomy) at a hospital in February 2012 and was told that there was no abnormality at the one-year check-up after the surgery. In November 2013, his physical examination revealed an abnormality in the remaining stomach, which was diagnosed as a recurrence of the remaining stomach, and he underwent a total gastrectomy in January 2014. In January 2014, he had a total gastrectomy. The pathological examination of the resected specimen showed that it was a scirrhous gastric cancer. He was told that the cancer was left at the anastomosis of the dissection. After his consultation, he was treated with intravenous lentinan (an immunostimulant) once a week and TS-1 capsules (an oral 5FU derivative, 20 mg) twice a month. Seven years later, in 2021, he is still alive and well.

(viii) Male, 68 years old (at time of first visit). Post-operative recurrence of gastric cancer; first visit to our hospital in May 2015.

In 2015, he went to the emergency room of his local doctor for hematemesis, was diagnosed as gastric hilar cancer and underwent emergency surgery.He underwent total gastrectomy and cholecystectomy and was diagnosed with anastomotic marginal gastric cancer remains. Postoperatively, tumor markers (CA72-4, CEA) were elevated, and recurrence was suspected.

**Figure 10:**
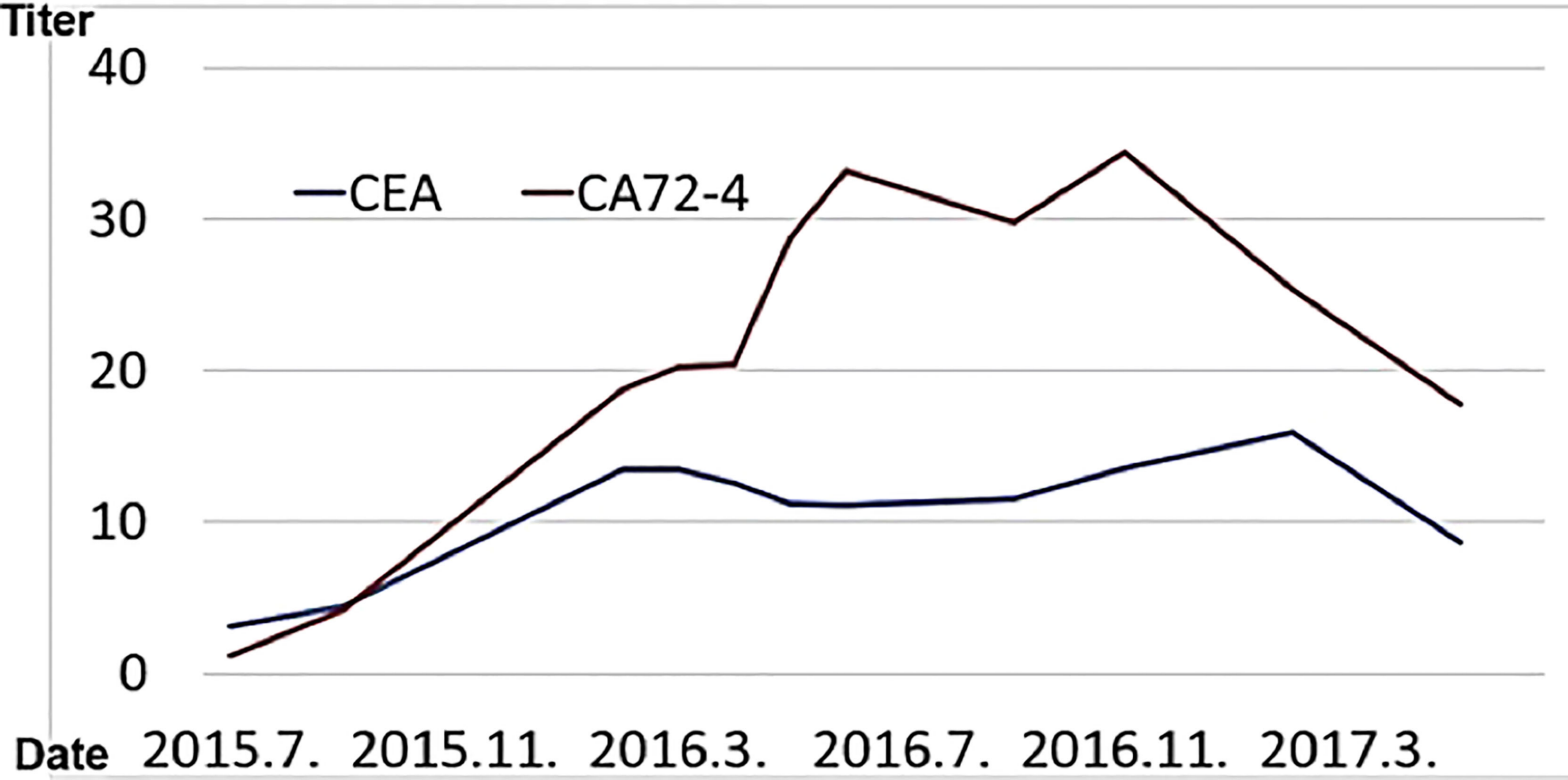
His tumor marker (CEA, CA72-4) values are shown.

In Wada clinic Lentinan (immunostimulant) was started intravenously and combined with TS-1 capsule (oral 5FU derivative, 20 mg) once a week. The patient is alive and well 6 years after surgery (**Figure 10**).

(ix) Female, 50 years old. Postoperative recurrence of breast cancer with multiple systemic metastases, including bone metastases. First visit to our clinic October 2019.

The resected tissue showed ER+, PgR+, HER2 1+ (negative), Ki67 26-29% (hormone receptor positive). In 2012, she underwent partial resection and lymphatic dissection for right breast cancer. In May 2020, a PET scan showed multiple metastases in the right axilla, vertebrae, and ribs, and she came to our clinic in October 2019. In terms of lifestyle, she loved sweets and enjoyed making pastries.

After her visit to our clinic, she has been on an ‘alkalizing diet’. In Wada clinic, we recommended her to do the following.

She took 140 ml/day of apple pectin juice (apple shimmer) at home, followed an alkalizing diet and ‘dairy products’ were strictly forbidden (79).

At the Wada Clinic she was given 25g vitamin C intravenously once a week. Subcutaneous injections of a luteinizing hormone-releasing hormone derivative microcapsule sustained-release formulation and a human IgG2 monoclonal antibody formulation targeting RANKL were continued.

**Figure 11:**
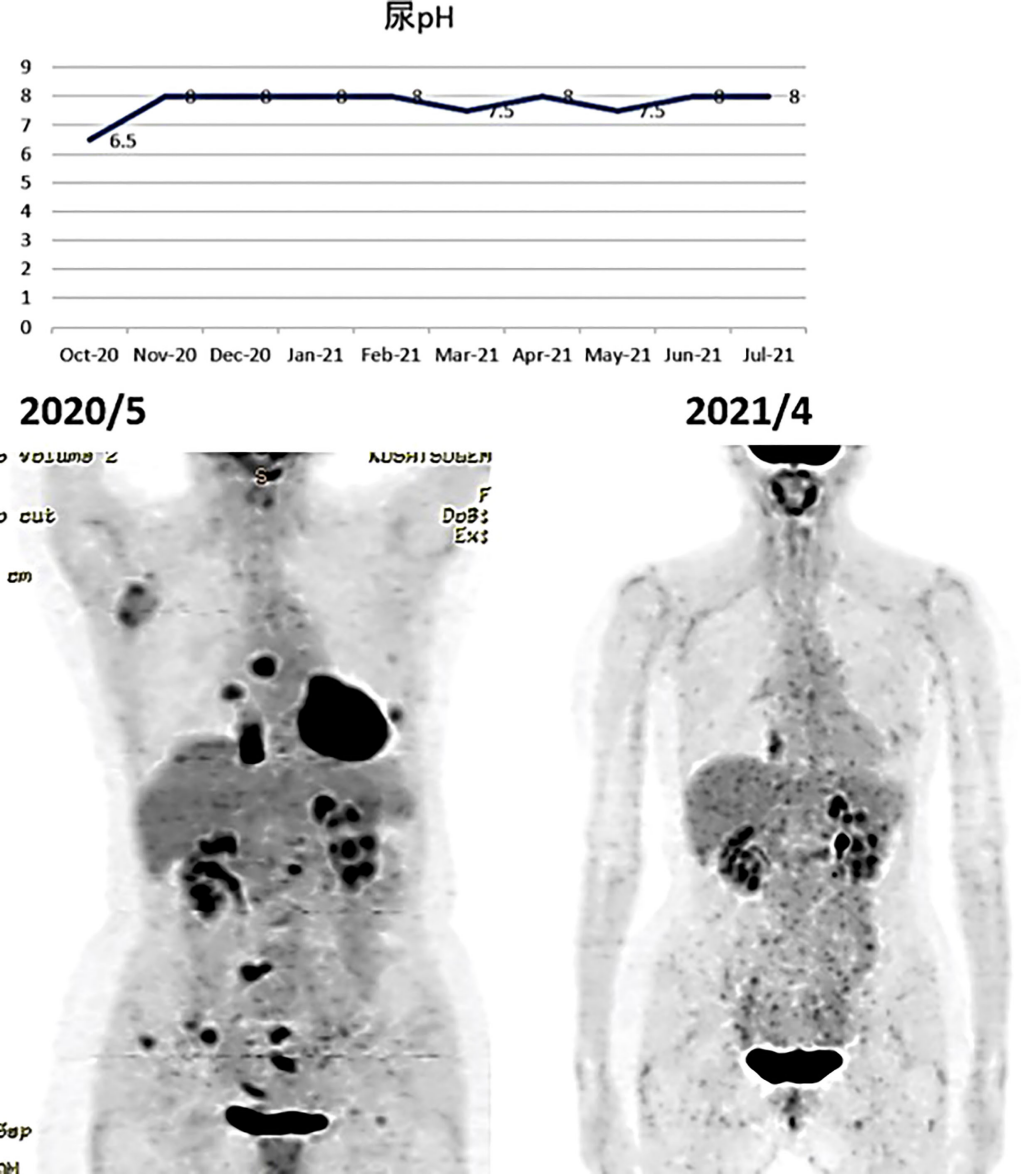
(Upper) Her urine pH levels are shown. (Lower) A PET/CT scan showed that tumor had disappeared.

A PET/CT scan six months after the first visit showed that the tumors had disappeared. The patient was also put on sodium bicarbonate and her urine pH increased from 6.5 on visit to 8.0 one month later (**Figure 11**).

(x) Female, 45 years old (at first medical examination). First visit to Wada Clinic in January 2011.

**Figure 12:**
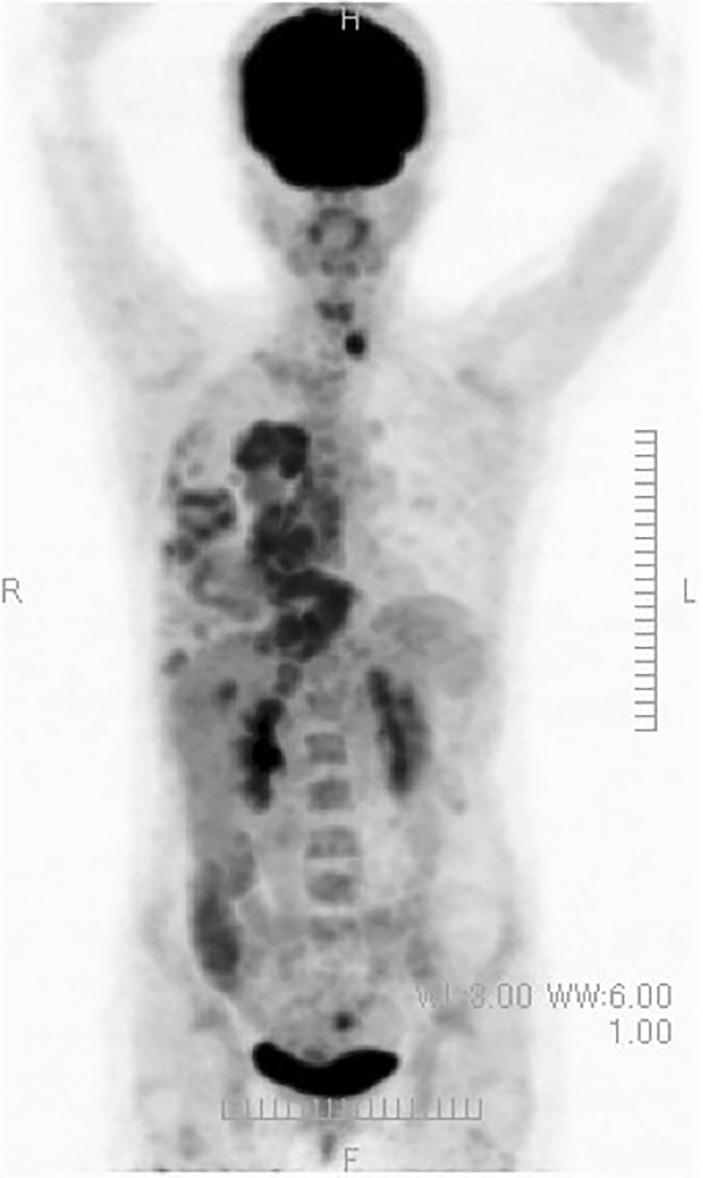
A PET/CT scan shows abnormal accumulations on her chest.

**Figure 13:**
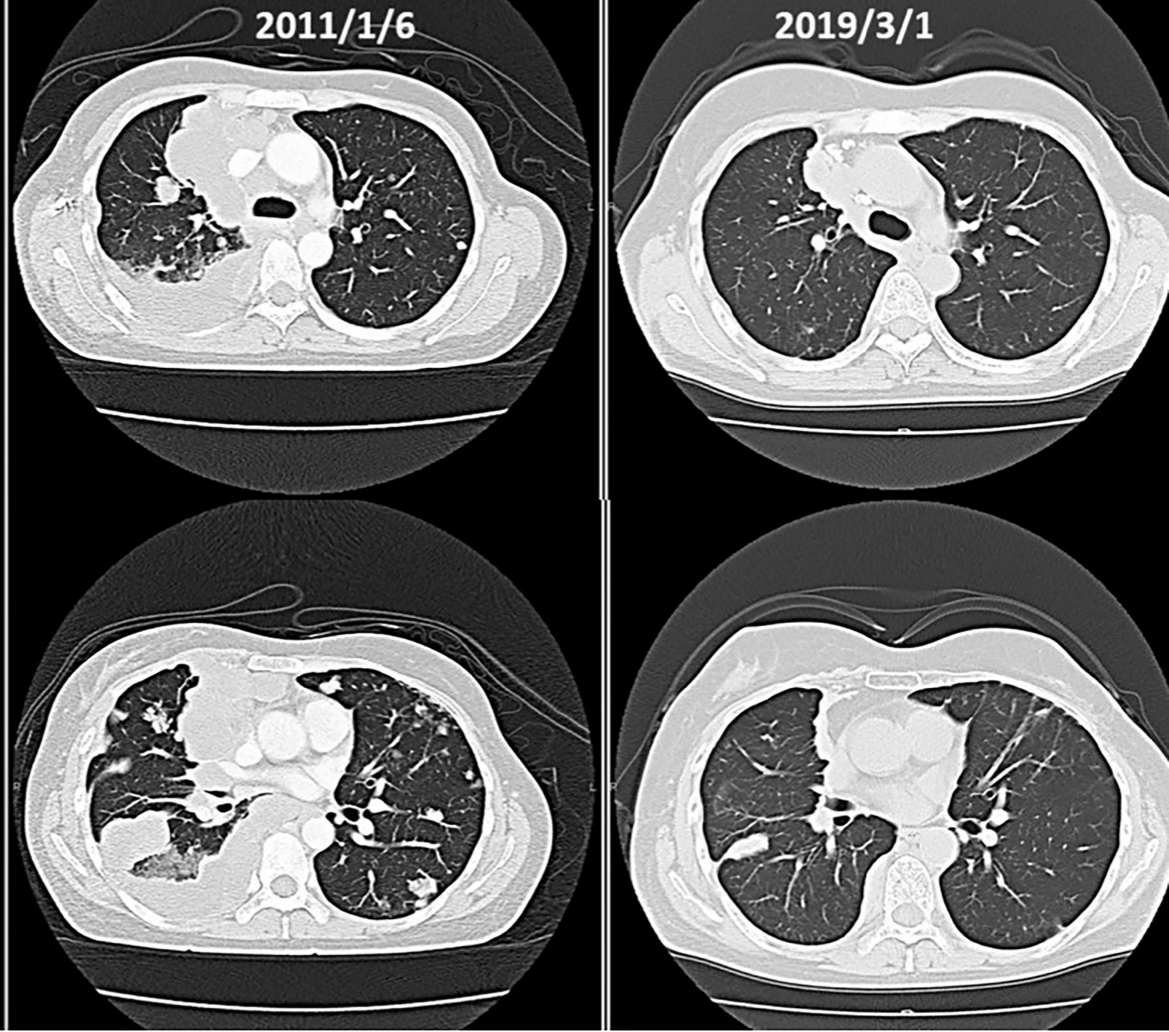
CT scans reveals gradual shrinkage of the tumors.

**Figure 14:**
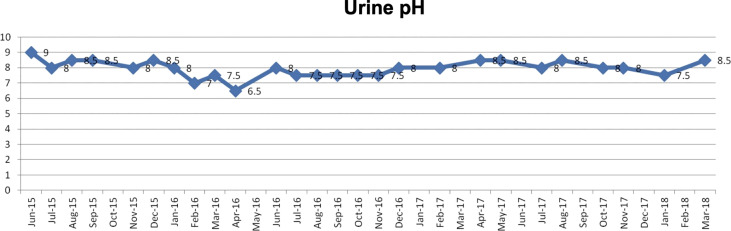
Her urine pH levels are shown.

Postoperative breast cancer, systemic metastasis including multiple lung and bone metastasis. Due to recurrent lung metastasis from breast cancer, she had breathing difficulties and started treatment at a cancer center, but the cancer did not shrink. After alkalizing diet, the cancer started to shrink (**Figure 12**, **13**). She has not received any anti-cancer drugs for more than 10 years. She drinks 2 liters of fruit and vegetable juice a day. In January 2022, when her urine is alkalized by diet alone (**Figure 14**), she is living her daily life in good health.

(xi) Female, 50 years old at the time of the first examination. Postoperative recurrence of breast cancer.

Breast cancer with axillary lymph nodes recurrence first visit of Wada Clinic on November 2016. She underwent breast cancer surgery in 2007 and had a recurrence in the right axillary lymph nodes (2014). We instructed her to eat an alkalizing diet, and had her take plume terpenes (Triterpenoids, Fatty acid synthase inhibitors), summer chrysanthemums (4) (Feverfew, NF kappa B inhibitors, parthenolide) and bicarbonate (3 g) (80–83). 85% regression was observed in spring 2017, and 100% regression and tumor disappearance in spring 2018.

## Data Availability Statement

The original contributions presented in the study are included in the article/supplementary material. Further inquiries can be directed to the corresponding author.

## Ethics Statement

The studies involving human participants were reviewed and approved by Japan-Multinational Trial Organization. Written informed consent to participate in this study was provided by the participants’ legal guardian/next of kin. Written informed consent was obtained from the individual(s), and minor(s)’ legal guardian/next of kin, for the publication of any potentially identifiable images or data included in this article.

## Author Contributions

HW and RH performed the literature review and wrote the article. RN and HM performed the acquisition of data. All Authors conceived and designed the study and gave final approval for publication.

## Conflict of Interest

The authors declare that the research was conducted in the absence of any commercial or financial relationships that could be construed as a potential conflict of interest.

## Publisher’s Note

All claims expressed in this article are solely those of the authors and do not necessarily represent those of their affiliated organizations, or those of the publisher, the editors and the reviewers. Any product that may be evaluated in this article, or claim that may be made by its manufacturer, is not guaranteed or endorsed by the publisher.
